# Novel Approaches for Treatment of Intraoral Microbial Infections

**DOI:** 10.1177/00220345251317494

**Published:** 2025-03-12

**Authors:** G. Hwang, Y. Liu, J. Korostoff

**Affiliations:** 1 Department of Preventive and Restorative Sciences, School of Dental Medicine, University of Pennsylvania, Philadelphia, PA, USA; 2 Center for Innovation & Precision Dentistry, School of Dental Medicine, School of Engineering and Applied Sciences, University of Pennsylvania, Philadelphia, PA, USA; 3 Chemical and Biomolecular Engineering, College of Engineering, Yonsei University, Seoul, Republic of Korea; 4 Department of Oral Health Sciences, Kornberg School of Dentistry, Temple University, Philadelphia, PA, USA; 5 Department of Periodontics, School of Dental Medicine, University of Pennsylvania, Philadelphia, PA, USA

**Keywords:** antimicrobials/antimicrobial resistance, bacterial virulence, biomaterial(s), infection control, infectious disease(s), alternative therapies

## Abstract

Historically, broad-spectrum antibiotics have represented a major component of the therapeutic armamentarium used to treat common oral diseases associated with a bacterial etiology. The fact that these diseases are due to the accumulation of multispecies biofilms composed of ever-increasing numbers of resistant organisms has dramatically affected the efficacy of many of these drugs. Furthermore, it is now appreciated that repeated use of broad-spectrum antibiotics also affects the composition of the host commensal microbiota, which can have both local and systemic implications. In recognition of the limitations of classical antibiotics, alternative chemical, physical, and mechanical strategies are either in use or development. These include novel narrow-spectrum antimicrobials such as antitoxins, bacteriophages, and antibody-conjugated drugs that can target specific microbes while minimizing the emergence of resistant organisms and preserving eubiotic microbes. Other approaches, such as new broad-spectrum non-antibiotic strategies and probiotics, are aimed at disrupting or altering the composition of oral biofilms and their extracellular matrices to facilitate the elimination of overt pathogens by the host response and/or adjunctive antimicrobials. This critical review describes the use and limitations of broad- and narrow-spectrum strategies currently being used to treat common bacterially induced oral diseases as well as alternative methods in development.

## Introduction

Historically, antibiotics have played a central role in the successful management of many bacterially derived infectious diseases, including oral diseases and conditions such as periodontitis and gingival/periodontal abscesses, among others. Most conventional antibiotics are bactericidal and typically function by disrupting protein or cell-wall biosynthesis or DNA replication and repair (Walsh 2000). Ironically, the use of the same molecules now threatens global human health due to the emergence of (multi)drug-resistant pathogens that are not susceptible to their antibacterial properties, necessitating the development of novel strategies for circumventing this issue. However, the complexity of “organ-specific” microbiomes, symbiotic/synergistic polymicrobial (or even cross-kingdom) interactions, the “protective” environment created by biofilms and extracellular matrices, and resultant dysbiosis therein renders an extremely challenging undertaking.

In particular, the oral cavity is considered the most complex microbial ecosystem in the human body, harboring a diverse multitude of bacteria and other microbes that collectively constitute its microbiome. The composition of the oral microflora is dependent on a variety of factors associated with microenvironments driven by diverse habitats, dynamic changes in diet, and interaction with the host in the oral cavity. Approximately 1,000 different microbial taxa are now known to be capable of colonizing oral tissues (Lamont et al. 2018), and many of these are critical to maintaining oral health. Perturbation of local environmental conditions can disrupt these symbiotic relationships, culminating in the outgrowth of endogenous pathobionts (dysbiosis) or accumulation of exogenous pathogenic species, potentially leading to disease onset. This is often associated with the breakdown of the barrier functions of epithelial and/or mucosal tissues that facilitate the transition from local to systemic pathology (Tanum et al. 2024). Notably, as shown for the oral–gut–liver, oral–gut–brain, and oral–lung axes, it is increasingly clear that the oral microbiome can influence conditions in other tissue compartments. Thus, the homeostatic state of the oral cavity achieved by the harmonious interplay of commensal bacteria with the host’s innate and adaptive immune responses is critical to maintaining local and systemic health (Zheng et al. 2020).

While antimicrobial resistance (AMR) comprises resistance to antibiotics by all types of microorganisms, antibacterial resistance is the most prevalent and significant in infectious diseases. Since this review focuses primarily on antibacterial resistance, we will use the term “AMR” specifically to refer to antibacterial resistance. Such AMR results from natural mutations within bacteria that can be passed between cells via several well-defined gene transfer mechanisms (Munita and Arias 2016). Over time, this provides a competitive advantage to resistant species, leading to their predominance over other microbiome members. In addition, antimicrobials affect not only pathogenic organisms but also commensals, resulting in reduced microbiome diversity. Collectively, the accumulation of resistant pathogens and the decrease in bacterial diversity can render the host vulnerable to other oral and nonoral infectious, as well as noninfectious diseases.

Unfortunately, developing new antibiotics that work via mechanisms similar to conventional antimicrobials currently in use is not a viable solution (Dolgin 2010). Hence, various new antibacterial strategies that function via alternative mechanisms are under development. This review summarizes the features of bacterially induced oral infectious diseases and the antimicrobial treatment modalities currently in use. We then narrate the characteristics of broad-spectrum and the most recent narrow-spectrum antibacterial strategies used to treat oral bacterial infections, as well as novel approaches under development as alternatives to classic antibiotics.

## Bacterially Induced Infectious Oral Diseases

**Figure 1.**
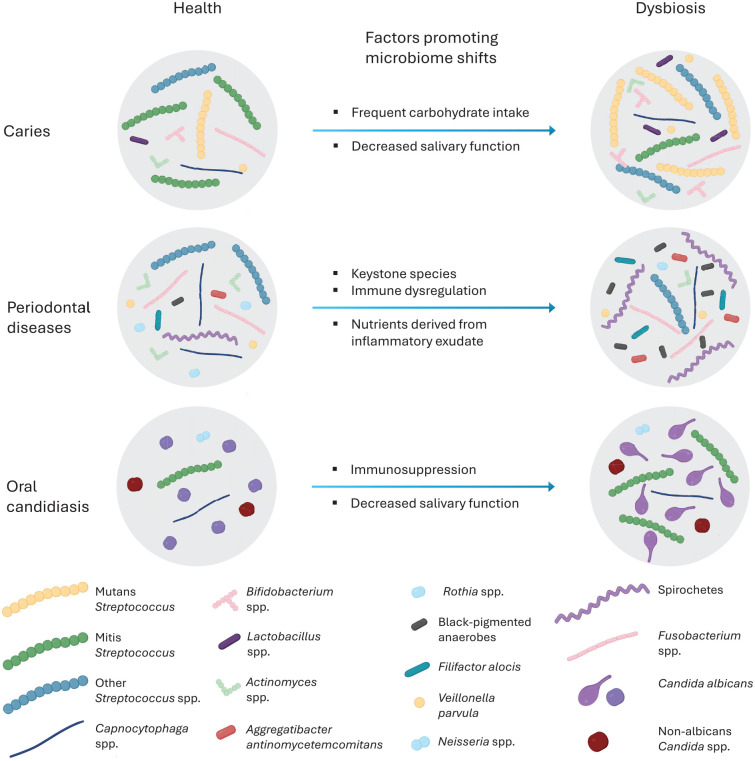
Dysbiotic changes associated with oral diseases. Oral diseases are linked to shifts in the microbiome community structure. Examples of these shifts, along with the main factors that promote the establishment of dysbiotic microbiota, are presented for conditions such as caries, periodontal disease, and oral candidiasis. Adapted from Hoare et al. (2017) with permission using Biorender.com.

The human oral cavity exhibits unique niches for bacterial growth and biofilm development due to diverse bacterial species and dynamic environmental conditions. Various habitats in the oral cavity that bacteria colonize (e.g., teeth, gums, tongue, and palate) vary in temperature, moisture, and oxygen levels. In addition, there are various types/amounts of consumed food and beverages in the oral cavity over time. All of these factors cause continual shifts in the composition of the oral microbiome. When homeostasis is disrupted, a disease-provoking microbiota can develop, ultimately resulting in dental caries, gingivitis, periodontitis, peri-implantitis, and oral candidiasis (Fig. 1). There are certain pathogenic bacteria uniquely involved in the specific diseases (e.g., *Streptococcus mutans* in dental caries and *Phorphyromonas gingivalis* and *Tannerella forsythia* in periodontitis). Although oral candidiasis is primarily a fungal infection characterized by *Candida* overgrowth, it often occurs when the bacterial balance is disrupted. In the following section, we will describe the characteristics of conventional broad-spectrum antibiotics for treating oral infectious diseases.

## Conventional Treatment of Bacterially Induced Oral Diseases

Mechanical biofilm removal has been the primary treatment modality for most bacterially induced oral diseases. Such approaches typically result in a reduction of the bacterial load, restoration of microbial balance, and resolution of inflammation. In contrast, antimicrobial agents are very rarely used to treat dental caries or periodontal disease, the most prevalent bacterially induced oral diseases. In certain cases, antiseptic mouth rinses (i.e., chlorohexidine) and topical or systemic antibiotics are administered as adjuncts to mechanical therapy. Most conventional antibiotics target bacterial cell membrane components or interrupt biosynthetic/metabolic pathways, resulting in the direct killing of the organisms (bactericidal) or suppression of their growth (bacteriostatic). Similarly, antibiotics commonly used to treat oral diseases work by inhibiting cell-wall biosynthesis (e.g., penicillins and cephalosporins) or protein biosynthesis (e.g., nitroimidazoles, tetracyclines, macrolides). These drugs tend to affect processes essential for bacterial proliferation or survival, with minimal specificity for target cells. AMR can develop through various mechanisms, such as alterations in bacterial membrane permeability, reducing antibiotics penetration into the cytoplasm, or actively discharging antibiotics via efflux pumps. Certain bacteria also can “disarm” antibiotics by modifying their target site or denaturing/degrading them in the cytoplasm (Cavaco et al. 2022). Notably, the normal lifestyle of oral microorganisms (i.e., formation of biofilms) significantly limits these conventional broad-spectrum antibiotics’ access to resident bacteria and minimizes their efficacies, potentially facilitating AMR development. Since bacteria that develop resistance to conventional antibiotics are most likely resistant to “newly” developed antibiotics that function via mechanisms similar to those of conventional broad-spectrum antimicrobials, novel antibacterial strategies employing alternative killing mechanisms are warranted. Specifically, alternative approaches should target pathogens without affecting the commensal community and maintain their efficacy when the organisms are living in multispecies biofilms.

## Narrow-Spectrum Antibacterial Agents

**Figure 2.**
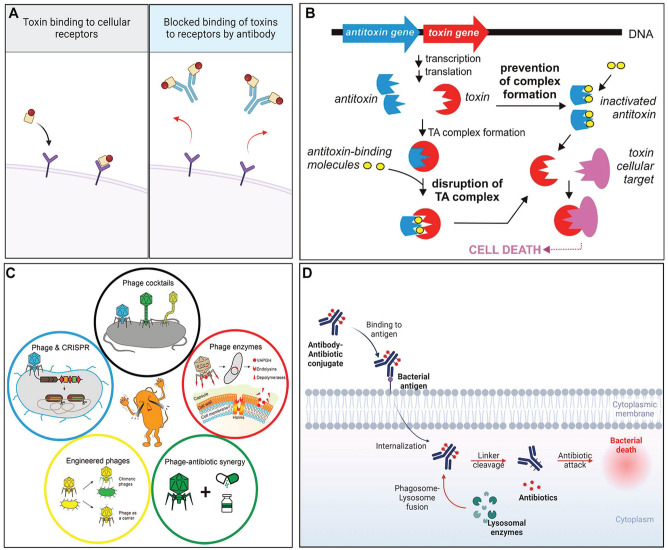
Narrow-spectrum antimicrobial strategies. (**A**) Inhibition of exotoxin binding to cellular receptors via antibody. Created with BioRender.com. (**B**) Disruption of the toxin–antitoxin system and/or prevention of toxin–antitoxin system formation. Adapted from Równicki et al. (2020) with permission. (**C**) Various strategies for phage therapy. Adapted from Wei, Peng, et al. (2020) with permission. (**D**) Diagram describing the mechanism of action of antibody–antibiotic conjugates. Created with BioRender.com.

To overcome issues associated with broad-spectrum antibacterial agents, a variety of narrow-spectrum antibacterial molecules with enhanced specificity and/or unique antibacterial mechanisms have been proposed. These include antitoxin antibiotics, bacteriophages, and antibody–antibiotic conjugates (Fig. 2). Although these narrow-spectrum antibacterials act via novel mechanisms, exhibit relative target cell specificity, and induce less resistance, they are not completely free from resistance development. Furthermore, the fate of narrow-spectrum antimicrobial agents is highly dependent on the rapid and reliable identification of pathogens before administration due to their target specificity. Despite their potent role, there have been very limited attempts to use these strategies in controlling oral infectious diseases. The features of recently developed narrow-spectrum antimicrobials that could be useful in treating oral bacterial infectious diseases are summarized in the following subheadings.

### Toxin-Targeted Antibacterials

An alternative strategy may involve specific interference of the synthesis or function of certain virulence factors that result in the neutralization of the disease-causing potential of the organism without the need to kill the pathogen. Exotoxins, a ubiquitous group of bacterial proteins that are either secreted or released when bacteria break apart, are involved in the pathogenesis of various infectious conditions (Sakari et al. 2022). While the normal oral microflora does not contain many exotoxin-producing bacteria, certain oral species (e.g., *Treponema denticola*, *Fusobacterium nucleatum*, and *Aggregatibacter actinomycetemcomitans*) (Silbergleit et al. 2020) and some external intruders (e.g., *Staphylococcus aureus*) (Hirose et al. 2021) are known to produce exotoxins that play a role in the pathogenesis of oral diseases. Therefore, impeding exotoxin activity has potential utility in treating these conditions in the oral cavity. This can be achieved by overtly inhibiting exotoxin synthesis or limiting the secretion of synthesized exotoxins. In addition, extracellular exotoxins can be inhibited by blocking downstream aspects of their effector mechanisms (Sakari et al. 2022). The most widely studied approach involves interfering with target cell binding through the application of intact or fragments of cell-binding domain-specific monoclonal antibodies (mAbs), receptor analogs, and neutralizing scaffolds (Fig. 2A). Although this approach might not be suitable for acute infections due to the amount of time required to identify the relevant exotoxin before initiating therapy, this strategy can still be beneficial when used in conjunction with relatively low concentrations of conventional antibiotics that kill pathogens but do not neutralize exotoxins, which may still be inflammatory.

Another toxin-based strategy involves targeting toxin–antitoxin systems that are prevalent in bacteria (Yang and Walsh 2017). In these systems, the toxins typically affect essential bacterial cellular processes, while the antitoxin component essentially maintains the toxin in a quiescent state. Activation of the toxin can therefore be deleterious to bacterial viability. Various approaches that aim to cause artificial activation of the toxin are being evaluated for their antibacterial efficacy, and it does appear that induced partial/complete degradation of the antitoxin in the toxin–antitoxin system can result in bacterial growth inhibition or even death (Fig. 2B) (Równicki et al. 2020). Although controversial, one potential drawback to targeting the toxin–antitoxin system is that, in certain bacteria, the activation of toxins may facilitate the generation of persister cells (Yang and Walsh 2017).

### Bacteriophages

Bacteriophages are prokaryotic viruses that are abundant in various environments. In particular, the oral cavity is densely populated by bacteriophage communities, which affect the ecology of oral bacterial communities and drive their genetic evolution (Edlund et al. 2015). Bacteriophages bind to receptors on the target bacterial surface, exhibiting high specificity up to strain levels. When in the lytic cycle, bacteriophages become virulent by replicating themselves using the bacterium’s genetic machinery, eventually resulting in the host cell bursting. Subsequently, released bacteriophages gain entry into uninfected bacteria and repeat this cycle, acting as self-amplifying drugs (Kortright et al. 2019).

Therefore, the use of bacteriophages has been suggested as a potent alternative antimicrobial strategy, especially for nosocomial and multidrug-resistant pathogens (Kulshrestha et al. 2024). Numerous clinical trials are currently being conducted worldwide to implement phage therapy (Petrovic Fabijan et al. 2023). Recently, the effectiveness of some oral pathogen–specific bacteriophages has also been explored, including *Fusobacterium* phage FNU1 (Kabwe et al. 2019), *Enterococcus* phage SHEF2 (Al-Zubidi et al. 2019), and numerous *Streptococcus* phages (Szafrański et al. 2021). Importantly, particular bacteriophages exhibit enhanced capabilities to penetrate oral biofilm extracellular matrices compared with conventional antibiotics. This enhanced penetration can potentially improve the efficacy of treating oral infections. However, several technical hurdles impede the progress of their therapeutic use, including the lack of clear guidelines for bacteriophage manufacturing (Mutti and Corsini 2019), the relative instability of produced bacteriophages, limited activity in biofilms, as well as a deficiency in rapid and high-throughput bacteriophage identification methods (Pires et al. 2020). Furthermore, similar to broad-spectrum antimicrobials, bacteriophages can induce resistance due to inevitable bacterial evolution. Potential mechanisms include prevention of bacteriophage binding, limitation of bacteriophage DNA insertion, degradation of phage DNA, disruption of bacteriophage replication/transcription/translation, and the development of anti-bacteriophage signaling systems (Bernheim and Sorek 2020). Bacteriophages may also activate bacterial toxin–antitoxin systems that can attenuate their replication pathway (Alawneh et al. 2016). To overcome these limitations, new phage therapies are currently under investigation, including phage cocktails, phage-driven enzymes, hybrid phage–antibiotics treatments, phage–CRISPR-Cas systems, and engineered phages (Fig. 2C). In addition, given the importance of maintaining a healthy microbiota relative to preventing the outgrowth and accumulation of endogenous and exogenous pathogens, bacteriophages can be used to engineer the composition of a microbiome by selectively controlling the population of targeted bacteria (Hsu et al. 2020).

### Antibody–Antibiotic Conjugates

Another promising alternative to conventional antibiotics is the use of antibody–antibiotic conjugate (AAC). AAC has three building blocks: an antibiotic payload that kills bacteria, an antibody that targets the payload delivery to the bacteria, and a linker that attaches the payload to the antibody and facilitates its release (Mariathasan and Tan 2017). In this way, AACs can deliver the drug directly to the surface of the target bacteria via binding of the mAb to a unique bacterial antigen, eventually killing them (Fig. 2D) (Cavaco et al. 2022). To date, AACs have been evaluated mainly in the context of nonoral bacterial infections, most notably *S. aureus*, and shown to exhibit efficacy in both animals and humans (Peck et al. 2019). Recently, it was reported that a *P. gingivalis*–specific mAb conjugated to ginsenoside Rh2, a broad-spectrum bactericidal phytochemical, selectively targeted the organism in the oral microflora of infected rats (Chen et al. 2023). In addition, the conjugate provided partial protection against gingival inflammation and alveolar bone loss in the rat model. Although promising, the use of such conjugates must be validated in additional animal studies and, eventually, human clinical trials. Unfortunately, AACs are not impervious to the development of resistance when used in a long-term manner. Given the high target specificity of AACs, pairing mAbs with novel alternative antimicrobials not prone to the induction of resistance should lead to more potent and selective therapeutic tactics for hard-to-treat infections. The use of humanized mAbs has reduced the immunogenicity of AACs relative to those containing animal-derived mAbs, further enhancing their efficacy as alternative antimicrobials.

## New Broad-Spectrum Nonantibiotic Strategies

In situations in which patients present with bacterial infections of unknown etiology, it is not possible to immediately determine the most appropriate narrow-spectrum antimicrobial agents. Under the time constraints required to identify the pathogen, certain infections, particularly acute ones, can progress rapidly and potentially become life-threatening if not treated effectively. Moreover, it has not been proven that eliminating a specific pathogen from the complex human oral microbiota will prevent or limit the progression of diseases such as periodontitis. Thus, new broad-spectrum antibacterial strategies have emerged that aim to maintain potent, broad-impact bactericidal activities but with distinctive killing mechanisms that might minimize the incidence of AMR. For example, oxidative stress–causing photodynamic therapy (PDT), immune-enhanced photobiomodulation therapy, and engineered antibiotics derivatized with various bioactive molecules such as nanoparticles are under development or in clinical use. In addition to chemical and biological tactics, ultrasonic vibration is under investigation as a physical form of broad-spectrum antimicrobial therapy. Each approach exhibits unique features, which are summarized in the following subsections.

### Phototherapies

**Figure 3.**
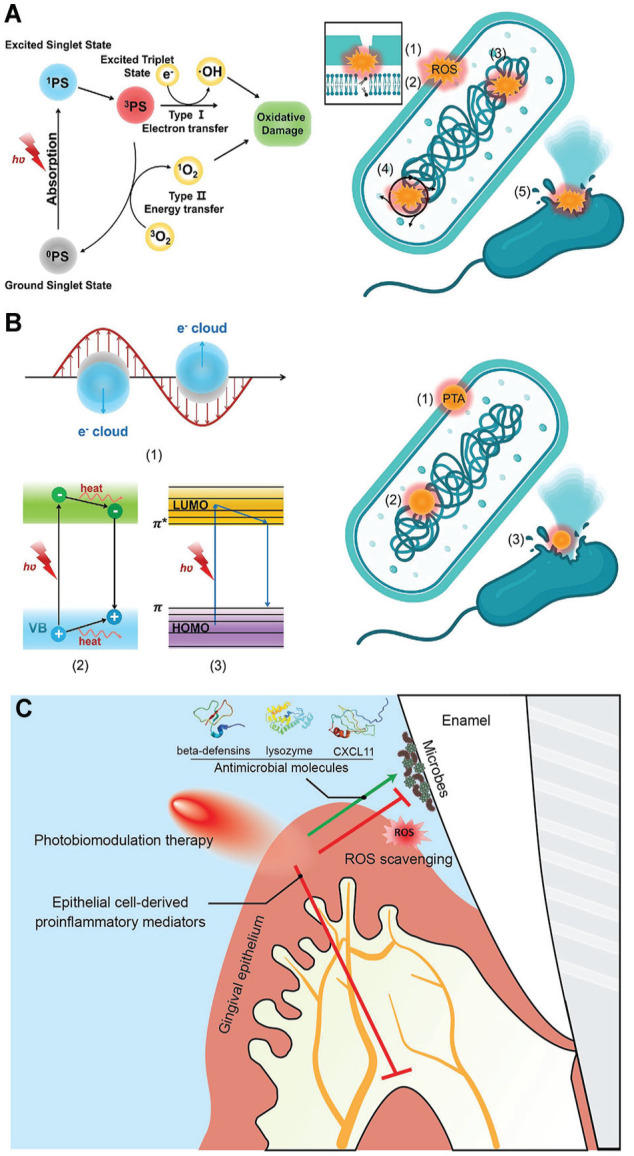
Mechanisms of phototherapies. Mechanism of (**A**) photodynamic therapy and its functions on bacteria illustrating (1) alteration of bacterial outer membrane permeability, (2) oxidation of lipids, (3) degradation of protein or DNA, (4) interference with bacterial metabolism, (5) permanent damage of bacterial envelope and (**B**) photothermal therapy and its functions on bacteria illustrating (1) alteration of bacterial outer membrane permeability, (2) denaturation of proteins, (3) permanent damage of bacterial envelope. Adapted from Huang, Qi, et al. (2023) with permission. (**C**) A schematic diagram describing the role of photobiomodulation therapy on the interactions between bacteria and gingival epithelium cells. Adapted from Tanum et al. (2024) with permission.

Since the concept of light therapy was introduced more than a century ago, a diverse array of diagnostic and therapeutic devices using light have been developed for clinical practice (Wei, Yang, et al. 2020). Two forms of phototherapy have been evaluated for their direct effect on microbial viability, PDT and photothermal therapy (PTT), which exhibit distinct mechanisms of action. PDT kills bacteria via the generation of reactive oxygen species (ROS) when a photosensitizer is activated by light either through type I mechanisms (electron transfer) or type II mechanisms (energy transfer) (Fig. 3A) (Huang, Qi, et al. 2023). In contrast, PTT damages bacteria via plasmonic heating, electron-hole generation, nonradiative relaxation, or thermal vibration of molecules (Fig. 3B) (Huang, Qi, et al. 2023). Both phototherapies are minimally invasive and exhibit significantly less chance of inducing AMR (Nguyen et al. 2022). Such phototherapies have been used in dentistry to treat oral soft- and hard-tissue conditions, including periodontitis, peri-implantitis, oral candidiasis, and jawbone infections (Huang, Qi, et al. 2023). PDT has also been shown to inhibit the growth of various microorganisms associated with cariogenic biofilms, such as *S. mutans*, *Lactobacillus*, and yeast (Garcia et al. 2021). PTT, in combination with 1% sodium hypochlorite, has demonstrated superior performance in endodontic treatment (Duan et al. 2022). Each therapy has its pros and cons. PDT does not require oxygen for bactericidal activity, making it effective in anaerobic environments, unlike PTT. However, the heat generated by PTT can damage adjacent host cells/tissues. In addition, both therapies can potentially interfere with the formation of eubiotic dominant microbiota due to their broad-spectrum bactericidal activities. This issue can be mitigated by conferring specificity to photosensitizers via chemical modification. Another concern is the reduced efficacy resulting from the limited solubility of photosensitizers in water and/or the challenges in light penetration needed to induce photodynamic or photothermal effects within the deeper aspects of oral biofilms due to their complex extracellular matrices.

A third form of light therapy is photobiomodulation therapy (PBMT), a nonpharmacologic form of host-modulatory therapy. It has been used to address a variety of medical conditions to provide pain relief, reduce inflammation, and/or facilitate wound healing (Kim et al. 2020). PBMT is not inherently bactericidal; rather, it enhances antimicrobial immunity via effects on a variety of different host immune and stromal cells (Tanum et al. 2024). Studies demonstrated that low-intensity light triggers photochemical changes within cells, facilitating the production of antimicrobial molecules (e.g., beta-defensin, lysozyme, and CXCL11) while minimizing the generation of free radicals when gingival keratinocytes were subjected to viable oral microbes (Fig. 3C) (Tanum et al. 2024). Ongoing research is being conducted to further investigate the utility of PBMT to treat oral and extraoral microbially induced inflammatory conditions.

### Ultrasound-Driven Cavitation

**Figure 4.**
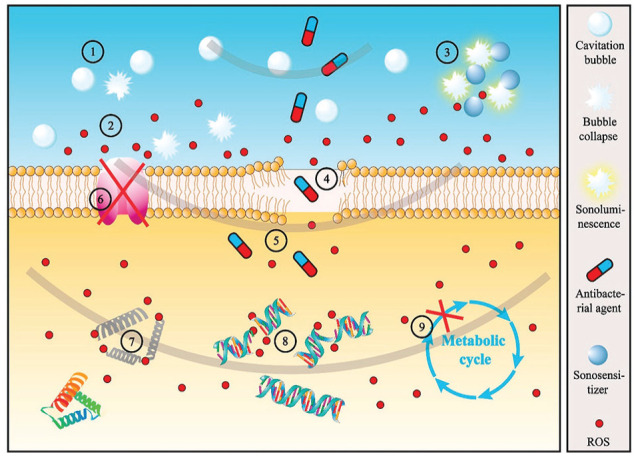
Schematic diagram illustrating the antibacterial mechanism induced by acoustic cavitation: ① formation and collapse of the cavitation bubble, ② reactive oxygen species (ROS) formation by sonochemistry, ③ ROS formation by sonoluminescence, ④ membrane perforation, ⑤ promotion of penetration of antibacterial agents, ⑥ transmembrane protein inactivation, ⑦ intracellular protein and enzyme inactivation, ⑧ DNA breakage, and ⑨ metabolism inhibition. Adapted from Dai et al. (2020) with permission.

Unlike most antibacterial strategies that rely on activated chemical molecules, ultrasound-driven cavitation minimizes the risk of developing resistant phenotypes. It achieves bactericidal effects mechanically through shock waves and shear stress as well as thermally (or chemically via locally generated ROS), by rapidly causing the collapse of microbubbles in an aqueous environment (Fig. 4) (Dai et al. 2020). Inducing such a bactericidal effect, however, may require a substantial amount of time (minutes to an hour) and energy, particularly when bacteria are encased within a protective biofilm matrix. In addition, the increase in local temperature and the resultant excessive amount of ROS can damage host cells/tissues. To address these issues, researchers have evaluated the synergistic effects of low-intensity ultrasound accompanied by antimicrobial peptides (Fitriyanti et al. 2023), plasma-loaded microbubbles (Zhu et al. 2023), and biofilm matrix-degrading enzymes (Lattwein et al. 2020). The outcome of these studies revealed enhanced bactericidal activities along with biofilm dispersion and surface removal. Indeed, removing surface-formed biofilms through the generation of cavitation bubbles and acoustic streaming is gaining attention in various medical and dental fields (Vyas et al. 2020). Given that acoustic cavitation can increase bacterial membrane permeability and perforate biofilm matrices to increase fluid flow, this technology has the potential to enhance the efficacy of both conventional and novel antibacterial strategies. In dentistry, this should be evaluated in the context of biofilm-associated conditions affecting root canals, the periodontium, and dental implants.

### Engineered Nanotechnology-Based Antibiotics

**Figure 5.**
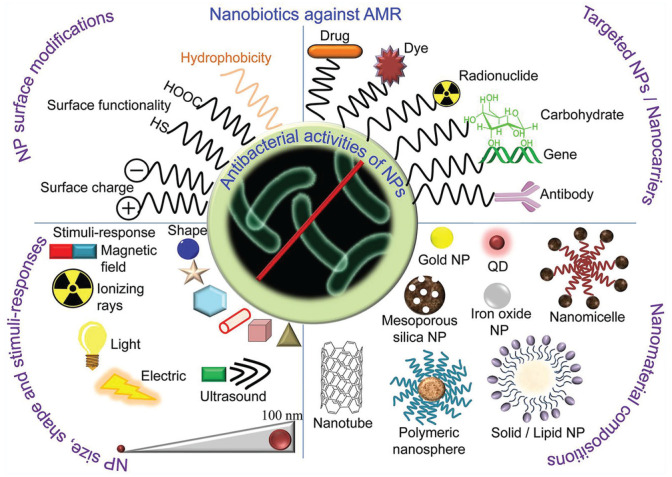
A variety of nanotechnology-based antimicrobial strategies. The unique small size of nanoparticles can enhance the interaction with bacteria due to the larger surface area–to–mass ratio. Nanoparticles are versatile, and their antibacterial activities can be controlled by modifying their size, shapes, and chemical compositions. Various types of nanoparticles can effectively destroy bacteria with multiple mechanisms, and their potency can be enhanced with the addition of ultrasound, magnetic field, light, and ionizing radiation properties. Adapted from Chakraborty et al. (2022) with permission.

Nanotechnology-based strategies comprise the most widely explored engineered approach to developing antimicrobial molecules with minimal risk of resistance. Certain nanomaterials have been used alone due to their intrinsic bactericidal activities, while others that do not affect bacterial viability are used as deliverables by carrying conventional antimicrobials. Among these, nanoparticles have been extensively used in dentistry as functional components in dental implants and fillings as well as for enamel surface polishing, caries prevention, and even tooth whitening (Mamidi et al. 2023). The mechanisms of various nanotechnology-based antibacterial strategies are illustrated in Figure 5, and these are detailed elsewhere (Chakraborty et al. 2022). The most common form of nanomaterials (i.e., nanoparticles) can be classified into three groups: metal/metal oxide-, polymer-, and carbon-based nanoparticles. The vast majority of bactericidal nanoparticles exhibit broad-spectrum activities but have distinct mechanisms of action compared with conventional antimicrobials. In particular, positively charged metal/metal oxide–based nanoparticles (e.g., silver, iron, titanium dioxide, cerium oxide) bind well to most bacterial surfaces via electrostatic interactions. This increases membrane permeability and/or disrupts vital intracellular processes through the release of metal ions or the generation of ROS (Zhang et al. 2022). Similarly, carbon-based nanoparticles (e.g., nanodiamonds, graphene) damage bacterial membranes/walls and/or induce oxidative stress through ROS-dependent or -independent mechanisms (Xin et al. 2019). Polymer-based nanoparticles, prepared from synthetic polymers or antimicrobial peptides, have been mainly used as carriers due to their physical/chemical stability and relatively high target specificity (Chakraborty et al. 2022). The drug-loading capability of these nanoparticles can enhance the efficacy of antimicrobials by preventing the loss of pharmacologic activity of insoluble drugs, preventing their degradation before encountering target bacteria, or increasing contact time. Based on fluoride’s ability to strengthen and remineralize tooth enamel, as well as inhibit bacterial acid production and plaque formation, fluoride-loaded nanoparticles are under investigation for the prevention and treatment of dental caries (Huang, Liu, et al. 2023).

Bolstered by the unique properties of nanoparticles, there have been attempts to create hybrid metal–polymeric nanoparticles that exhibit outstanding antimicrobial activities and enhanced compatibility with various materials (Delgado et al. 2011). In addition, some nanomaterials have been used in combination with other approaches, such as a photosensitizer for PDT or a sonosentitizer for ultrasound therapy. Furthermore, due to their excellent penetration properties, some nanoparticles are set to deliver molecules inside the host cells to modulate their immune system response (Bahlool et al. 2022). Given the fact that the release of loaded drugs within nanoparticles can be triggered by various external and internal stimuli (e.g., light, magnetic field, pH, enzyme), there are many avenues for the development of novel antimicrobial nanomaterials. However, the cytotoxic potential of nanoparticles must be thoroughly evaluated prior to clinical application. Biodegradable nanoparticles might minimize the accumulation of spent nanomaterials in the human body. In addition, bacterial adaptions resulting in resistance to nanomaterials have been reported (Xie et al. 2023). Finally, some challenges in the manufacturing process, such as variability in the size of nanomaterials, particularly for polymer-based nanomaterials, require further research.

## Probiotics

In dentistry, the use of probiotics is an emerging approach to managing oral health. By intentionally introducing beneficial bacteria to the complex oral microbiota, the growth of pathogenic bacteria can be suppressed, restoring microbial balance (Hoare et al. 2017). This likely occurs via several mechanisms. Particular probiotics contain bacteria that outcompete pathogens/pathobionts for resources by producing antimicrobial substances, modulating the host’s immune response, or producing bacteriocins. *Lactobacillus* and *Bifidobacterium* species are commonly used to control the population of *S. mutans* (Luo et al. 2024), while *Lactobacillus reuteri* has been shown to be effective in reducing the levels of periodontal pathogens, including *P. gingivalis* and *Aggregatibacter actinomycetemcomitans* (Agossa et al. 2022). In addition, probiotic therapy has been shown to be effective in moderating halitosis and peri-implantitis (Amato et al. 2022). Unique delivery strategies are under development, involving the supplementation of dental products with probiotics. These include chewing gum, mouth rinses, and toothpaste, which may provide prolonged contact time for enhanced efficacy. While some reports have demonstrated the effectiveness of probiotics in reducing pathogenic bacteria and inflammation in the mouth without causing AMR, it is unlikely that they can fully replace standard oral hygiene practices due to their limited efficacy and the dynamically changing oral microenvironment.

## Summary and Outlook

The use of broad-spectrum antimicrobials to treat oral conditions associated with a bacterial etiology is well established, especially as an adjunct to the mechanical disruption of biofilms. However, due to the rising incidence of AMR, the complexity of the oral microflora, the propensity of oral bacteria to exist in biofilm matrices, and the microbial/physiological interactions between the oral cavity and other tissues, the development of alternatives to conventional antibacterial strategies is warranted. There is ample evidence showing that certain oral bacteria can be transmitted to various parts of the human body, such as the gut (Schmidt et al. 2019) and brain (Roy et al. 2023), resulting in perturbation of the tissue-specific microbiota and the onset of nonoral disease. Thus, retaining eubiotic microbiota in the oral cavity without causing AMR is vital to the development of host defense mechanisms and/or suppression of the emergence of pathobionts, potentially limiting the occurrence of oral and systemic diseases. Although there are some studies demonstrating that narrow-spectrum antimicrobial strategies are clinically as effective as conventional broad-spectrum antimicrobials when evaluated in children (Demeke et al. 2021), adults (Tracy et al. 2022), and elderly patients (Joyner et al. 2022) treated for different infectious diseases, it has not been fully proven that eradicating that specific pathogen will attenuate the progress of oral infectious diseases. Furthermore, it appears that some narrow-spectrum and alternative broad-spectrum antimicrobial strategies still possess the potential to develop resistance mechanisms, albeit at a much slower pace. It is therefore imperative that the use of conventional antibiotics be under strict control to tame the global AMR crisis until novel, innovative approaches emerge.

Especially relevant to dentistry, it is critical to consider microbial interactions during the pathogenesis of certain diseases when evaluating the efficacy of novel strategies. Oral bacteria are often contained within multispecies biofilms and even interact with nonbacterial organisms. For example, the coexistence of the biofilm-forming cariogenic bacterium *S. mutans* and the opportunistic fungus *Candida albicans* is relevant to the etiology of early childhood caries (Hwang 2022). While an effective treatment measure is urgently needed to manage this condition, conventional approaches have not been efficacious because most do not affect bacteria and fungi simultaneously and/or are not appropriate for use in children. To directly target the bacterial-fungal interactions, tactics focused on dissociating their synergistic interaction by using enzymes instead of killing them show promise (Kim et al. 2021). Notably, expanding the scope of antimicrobial approaches from targeting single bacterial cells in their planktonic state to addressing community issues, such as dismantling or removing oral biofilms and complex matrices composed of various extracellular substances, will be beneficial (Karygianni et al. 2020; Jakubovics et al. 2021). This will require the development of not only novel chemical-based approaches but also strategies that function through physical/mechanical mechanisms.

## Author Contributions

G. Hwang, contributed to conception, design, data acquisition, analysis, and interpretation, drafted and critically revised the manuscript; Y. Liu, J. Korostoff, contributed to acquisition, analysis, and interpretation, critically revised the manuscript. All authors gave final approval and agree to be accountable for all aspects of the work.
